# A Sensitive, Portable Microfluidic Device for SARS-CoV-2 Detection from Self-Collected Saliva

**DOI:** 10.3390/idr13040097

**Published:** 2021-12-14

**Authors:** Jianing Yang, Mark Kidd, Alan R. Nordquist, Stanley D. Smith, Cedric Hurth, Irvin M. Modlin, Frederic Zenhausern

**Affiliations:** 1 Center for Applied NanoBioscience and Medicine, College of Medicine-Phoenix, University of Arizona, Phoenix, AZ 85004, USA; arnordqu@arizona.edu (A.R.N.); sdsmith7@arizona.edu (S.D.S.); churthua@gmail.com (C.H.); 2 WREN Laboratories, Branford, CT 06405, USA; mkidd@wrenlaboratories.com (M.K.); imodlin@irvinmodlin.com (I.M.M.)

**Keywords:** SARS-CoV-2, saliva, microfluidics, cartridge, integration, detection, sensitivity

## Abstract

Since the outbreak of the severe acute respiratory syndrome coronavirus 2 (SARS-CoV-2) pandemic in December 2019, the spread of SARS-CoV2 infection has been escalating rapidly around the world. In order to provide more timely access to medical intervention, including diagnostic tests and medical treatment, the FDA authorized multiple test protocols for diagnostic tests from nasopharyngeal swab, saliva, urine, bronchoalveolar lavage and fecal samples. The traditional diagnostic tests for this novel coronavirus 2019 require standard processes of viral RNA isolation, reverse transcription of RNA to cDNA, then real-time quantitative PCR with the RNA templates extracted from the patient samples. Recently, many reports have demonstrated a direct detection of SARS-Co-V2 genomic material from saliva samples without any RNA isolation step. To make the rapid detection of SARS-Co-V2 infection more accessible, a point-of-care type device was developed for SARS-CoV-2 detection. Herein, we report a portable microfluidic-based integrated detection-analysis system for SARS-CoV-2 nucleic acids detection directly from saliva samples. The saliva cartridge is self-contained and capable of microfluidic evaluation of saliva, from heating, mixing with the primers to multiplex real-time quantitative polymerase chain reaction, detecting SARS-CoV-2 with different primer sets and internal control. The approach has a detection sensitivity of 1000 copies/mL of SARS-CoV-2 RNA or virus, with consistency and automation, from saliva sample-in to result-out.

## 1. Introduction

Severe acute respiratory syndrome coronavirus 2 (SARS-CoV-2) and its associated infectious disease has swept the world quickly and become a global pandemic since its outbreak in December 2019 (COVID-19). The significant morbidity, mortality and transmission of COVID-19 caused unprecedented demand for rapid diagnostic testing to screen large populations of the public. The FDA approved multiple test protocols under an emergency use authorization (EUA) to provide more accurate and timely access to medical countermeasures [1]. Currently, there are two different types of COVID-19 viral infection testing authorized, i.e., molecular diagnostics (e.g., PCR) and antigens tests. Due to the high false-negative rate, antigen detection test is preferably used only for excluding those patients with high viral loads [2]. As for an indirect test for SARS-CoV-2, a serological test to detect antibodies to SARS-CoV-2 viral coat protein (e.g., spike protein) has been performed in clinical and research laboratories, with methods including enzyme-linked immunosorbent assay (ELISA), chemiluminescence, Western blotting, etc. However, its implementation is limited by the longer timeline, higher cost and limited ability to scale-up to larger throughput.

Direct molecular diagnostic testing (MDx) plays a critical role in the global response to COVID-19 pandemic and is achieved by nucleic acid amplification tests (NAATs) based on the genomic sequence of SARS-CoV2 virus. It begins with: (i) isolation of viral RNA from samples collected from patient’s nasopharyngeal (NP) swabs, sputum, blood, feces, urine or bronchoalveolar lavage, and (ii) conversion of viral RNA to complementary DNA (reverse transcription, RT), followed by (iii) real-time polymerase chain reaction (qPCR) [3,4]. Currently, this RT-qPCR remains the gold standard among all diagnostic tests for SARS-CoV-2 detection. Based on nanotechnology, RT loop-mediated isothermal amplification (RT-LAMP) and a colorimetric assay with Au nanoparticles (AuNPs) conjugated with thiol-modified antisense oligonucleotide primer have also been developed into a portable platform of SARS-CoV-2 testing [4]. For the diagnosis of SARS-CoV-2 infections, covering the distribution of SARS-CoV2 virions in body fluid and tissues, different detection methods and diagnostic assays were developed and reported into systemic literature reviews [2,4].

In response to the increased demand for rapid accurate testing, efforts have been deployed for designing a simplified SARS-CoV-2 viral RNA isolation protocol [5] and selection of more sensitive RT-PCR kits [6] have progressed. As for a non-invasive method of sample collection, NP swabs were originally considered as a standard sample source for COVID-19 molecular testing. However, the requirement of trained personnel to perform the NP swab collection limits its consumer availability. In a systemic review of meta-analysis from the MEDLINE and medRxiv database, saliva and NP swab nucleic acid amplification testing (NAAT) for SARS-CoV2 detection were compared [7]. The results showed that the saliva NAAT pooled sensitivity was 83.2% and pooled specificity was 99.2%, while NP swab NAAT had a sensitivity of 84.8% and a specificity of 98.9%. There was no statistically significant difference. Many reports suggested that saliva specimen is an excellent alternative biofluid sample for SARS-CoV-2 detection [8,9,10]. Although it showed lower viral load than NP swab specimens, saliva samples provide several advantages in SARS-CoV-2 specimen collection, such as being non-invasive and convenient for self-collection, it has less patient discomfort, large specimen volumes can be collected, and it provides an ease-of-use amongst other parameters. It also provides comparable sensitivity to NP swabs [8,10,11,12,13]. Recently, the detection of SARS-CoV-2 was compared between saliva and NP or OP swab samples in a meta-analysis [14]. This conclusively demonstrated that the saliva was as valid as nasopharyngeal sampling for the detection of SARS-CoV-2 infection in both symptomatic and asymptomatic carriers.

The standard diagnostic assay for SARS-CoV-2 recommended by the Center for Disease Control and Prevention (CDC) and the World Health Organization (WHO) is composed of two steps of (1) viral RNA extraction from the specimen and (2) reverse transcription-quantitative polymerase chain reaction (RT-qPCR). Due to the time and labor required for an RNA extraction step, and world-wide shortage of reagents supply for RNA extraction, many research groups began testing direct RT-qPCR detection of SARS-CoV-2 RNA from either NP swabs or saliva samples, bypassing the RNA extraction step [15,16,17,18,19,20]. For patient NP swab samples stored and transported in viral transfer media (VTM), SARS-CoV-2 RNA was successfully detected directly from the termed diluent without an RNA extraction step [15]. However, the detection sensitivity dropped about 4 Ct values (2^4^ = 16-fold) when compared to the same pooled NP swab diluent with the RNA extraction step. The addition of a heating step (95 °C for 10 min) prior to direct RT-qPCR improved the sensitivity, especially for low viral copy samples [15]. In another study of 597 clinical patient NP swab samples, heat-inactivation time between 5 min to 15 min incubation was quite stable in the extraction-free protocol [16]. Several reports of saliva-based SARS-CoV-2 testing that bypasses RNA isolation/purification were demonstrated [18,19,20]. SARS-CoV-2 detection from saliva, without the viral isolation step, was shown to be a simple and sensitive molecular diagnostic test [18,20]. Many protocols were examined by using different dilution buffers (e.g., TBE, TE, or PBS) or adding additives (e.g., Triton-X-100, Tween-20, or NP-40), and the limit of detection (LOD) was compared to standard protocol with viral RNA isolation before RT-qPCR [18]. The optimized and simplest protocol was the addition of TBE (or TE) buffer at a 1:1 ratio with saliva followed by heat treatment at 95 °C for 30 min and addition of Tween-20 to a final concentration of 0.5%. Comparable LOD results were reported between direct saliva-to-RT-qPCR and RNA extracted from a saliva specimen, with a sensitivity of about 5000 viral copies/mL of γ-irradiated SARS-CoV-2 spiked in saliva. Another study using the SalivaDirect^TM^ protocol demonstrated that by mixing proteinase K with the saliva sample and heat inactivation at 95 °C for 5 min, a duplex RT-qPCR could detect as low as 6–12 copies/µL [20].

To address the high demand for rapid testing, several non-conventional, chip-based assay platforms were also developed, targeting for rapid SARS-CoV-2 diagnosis. For example, a nanomaterial-based (gold nanoparticles—AuNPs) technology was reported for SARS-CoV-2 detection with a colorimetric bioassay [21] or the lateral flow assay (LFA) [22]. Using CRISPR-based nuclease cleavage assay to detect SARS-CoV-2 was also developed [22,23]. Recently, a group of engineers developed a stamp-sized microfluidic chip which quantitatively detects SARS-CoV-2 nucleocapsid (N) protein spiked in human serum sample [24]. It was reported that this handheld smartphone-based immunosensor device could detect as low as 50 pg/mL of SARS-CoV-2 N protein in a whole serum specimen in less than 60 min. As for a point-of-care (POC) assay development, a CRISPR-based POC diagnostic, miSHERLOCK platform was reported, capable of testing SARS-CoV-2 variants from a saliva sample [25]. The built-in saliva preparation used an RNA paper-capture method, followed by one-pot SHERLOCK reaction of isothermal amplification and Cas-mediated detection, achieving a LOD of 1000 copies/mL. A cartridge-based, real-time RT-PCR or CovidNudge test was developed by DnaNudge, which requires no sample pre-processing and no laboratory handling [26] for COVID-19 testing using their handheld NudgeBox analyzer. Comparing to standard laboratory RT-qPCR, the CovidNudge POC platform demonstrated overall sensitivity and specificity of 94% and 100%, respectively. Another portable, field-deployable microfluidic device for COVID-19 infection and other disease-causing organisms, with a teardrop amount of sample input from NP swab was reported [27]. There are many commercial companies that have developed small, rapid SARS-CoV-2 test systems and received EUA approval [28]. For example, the Accula^TM^ System (Mesa Biotech, San Diego, CA, USA) developed a cartridge-based, rapid immunoassay system, which detects SARS-CoV-2 quantitatively in 30 min with visually read results [29]. Abbott’s ID NOW^TM^ system (Abbott, Lake Forest, IL, USA) uses isothermal nucleic acid amplification technology. It can process NP swabs and saliva samples and provide the test result in 15 min [30]. The Cepheid Xpert Xpress SARS-CoV-2/Flu/RSV system (Cepheid, Sunnyvale, CA, USA) can provide rapid detection of current coronavirus SARS-CoV-2 in 25 min for positive results and provide results for four pathogens in 36 min, with less than a minute hands-on time [31]. Moreover, an open-source design of a 3 D-printed centrifuge device, Mobilefuge, was developed for SARS-CoV-2 testing from saliva samples, using Loop-Mediated Isothermal Amplification (LAMP) and can be powered from USB ports and even a mobile phone [32]. Although there have been many POC devices for SARS-CoV2 testing reported, the usage of only the purified viral RNA or single-plex amplification was demonstrated. In addition to high manufacturing cost, most of the POC devices reported emphasized the speed of their analytical process, rather than emphasizing the sensitivity of the test. They often omitted the sample preparation time in their estimation of the overall test duration.

Here in, we report a sensitive, portable, microfluidic-based integrated detection-analysis system (MiDAS) for the direct detection of SARS-CoV-2 from saliva specimens. We rapidly adapted our existing microfluidic-based integrated rapid DNA analysis system, originally developed for DNA forensic genotyping and other pathogen detection applications [33,34] to the testing for SARS-CoV-2. The reconfiguration of the system demonstrates the concept of “saliva sample-in and multiplex amplification signals-out” with real-time monitoring. We achieved a sensitivity of nine copies per reaction volume (45 mL) for on-cartridge RT-qPCR detection, which was equivalent to 1000 copies/mL of SARS-CoV-2 RNA in the input saliva specimen. This platform provides an automated portable, self-contained, microfluidic-based COVID-19 test system for sensitive detection from a self-collected saliva sample, which has a significant impact on public health emergency response, especially for pandemic screening in remote settings.

## 2. Materials and Methods

### 2.1. Collection and Preparation of Saliva Samples

Fresh saliva was collected from healthy volunteers, who were required to rinse their mouths with water and ingest no food or beverage for at least 3–4 h before collection. The saliva was collected in sterile 50 mL conical tubes (BD Falcon, Houston, TX, USA) based on University of Arizona College of Medicine-Phoenix IRB-approved protocol. Saliva was diluted with TBE buffer (100 mM Tris-HCl pH8.0, 90 mM boric acid, and 1 mM EDTA) at 1:1 ratio, as described [18]. Samples were then aliquoted into sterile 1.5 mL Eppendorf tube at 200 µL each and stored at −20 °C till use.

On the day of an experiment, known amounts of either 2019-nCoV N Plasmid-RNA (Integrated DNA Technologies (IDT), Coralville, IA, USA; Lot no. 10006625) or intact, γ-irradiated SARS-CoV-2 virions (BEI, Manassas, VA, USA; Cat# NR-52287, Lot no. 70039067) were spiked into saliva samples. Saliva samples were heated at 100 °C for 5 min in a water bath in-tube [18,20] or for 10 min on-cartridge. During the heating period evaluation, a time course was generated by heating saliva samples at 10 min, 5 min, 2 min, 1 min, or 0 min, respectively. The clinical SARS-CoV-2 RNA samples isolated from saliva and/or nasopharyngeal (NP) swabs were obtained from WREN Laboratory (WREN Laboratory, LLC; Branford, CT, USA) under a material transfer agreement (MTA).

### 2.2. RT-qPCR Amplification

We performed multiplex RT-qPCR assay using the TaqPath 1-Step Multiplex RT-qPCR Master Mix (Thermo Fisher Scientific, Carlsbad, CA, USA; Cat# A28525) and Qscript Lyo 1-Step Multiplex RT-qPCR (Quantabio, Beverly, MA, USA; Cat# 76312-730) during the development phase, and using Qscript Lyo 1-Step Multiplex RT-qPCR for the on-cartridge integration. Qscript Lyo 1-Step Multiplex RT-qPCR Master Mix is in a lyophilized pellet format and this solid-phase PCR Master Mix is easier for embedding into a cartridge chamber; it is also convenient for on-cartridge storage. COVID-19 PCR primers were custom synthesized by Integrated DNA Technologies (IDT), with selective fluorescein labels by WREN Laboratory. The 2019-nCoV_N1 primer-probe set and N3 primer set were employed to detect SARS-CoV-2 N viral genes. They were: 2019-nCoV_N1-F: 5′-GAC CCC AAA ATC AGC GAA AT-3′, N1-R: 5′-TCT GGT TAC TGC CAG TTG AAT CTG-3′, N1-P: 5′-FAM-ACC CCG CAT TAC GTT TGG TGG ACC-3′; N3-F: 5′-GGG AGC CTT GAA TAC ACC AAA A-3′, N3-R: 5′-TGT AGC ACG ATT GCA GCA TTG-3′, N3-P: 5′-ABY-ATC ACA TTG GCA CCC GCA ATC CTG-3′. The human RNase P (RNP) gene was used as an internal control. The RNP primer-probe set was: RP-F: 5′-AGA TTT GGA CCT GCG AGC G-3′, RP-R: 5′-GAG CGG CTG TCT CCA CAA GT-3′, RP-P: 5′-VIC-TTC TGA CCT GAA GGC TCT GCG CG-3′. The working concentration of each primer-probe set mixture contains 20 µM of forward and reverse primers, and 5 µM of probe.

The 1-Step RT-qPCR reaction master mix was prepared following the manufacturer’s protocol. For TaqPath 1-Step Multiplex RT-qPCR Master Mix, 1 µL of N1 and N3 primer-probe sets and 0.5 µL of RNP primer-probe set were added into the RT-qPCR reaction master mix, in a 20 µL of standard benchtop multiplex RT-qPCR reaction, while in the on-cartridge multiplex RT-qPCR, the volume of each primer-probe set added into the PCR reaction mix would be changed proportionally as the reaction volume changed. The final concentrations were kept the same for both in-tube or on-cartridge PCR reactions. The final concentrations in a RT-qPCR reaction mix were 1 µM of N1 or N3 forward and reverse primers, 500 nM of N1 or N3 probes, 500 nM of RNP forward and reverse primer with 250 nM of RNP probe. When using the lyophilized Qscript Lyo 1-Step Multiplex RT-qPCR Master Mix reagent, one lyophilized bead was used per RT-qPCR reaction, regardless of the assay platforms of in-tube or on-cartridge. The Qscript Lyo 1-Step Multiplex RT-qPCR bead was designed to be used for on-cartridge assay platform, while the TaqPath liquid-form 1-Step Multiplex RT-qPCR reagent was evaluated initially as a benchmark standard for the on-cartridge assay protocols development and evaluation.

The thermal cycling conditions were set according to manufacturer’s protocol. For the TaqPath 1-Step Multiplex RT-qPCR (Thermo Fisher Scientific, Carlsbad, CA, USA), 1 cycle of 2 min at 25 °C and 1 cycle of 10 min at 53 °C, were followed by a 2 min incubation at 95 °C for denaturation, then 40 cycles of 95 °C 15 s and 60 °C 1 min. For Qscript Lyo Multiplex 1-Step RT-qPCR (Quantabio, Beverly, MA, USA), the RT step was at 50 °C for 15 min. Following the initial denaturation at 94 °C for 3 min, 40 cycles of 2-step PCR cycling included 94 °C for 15 s and 60 °C for 1 min. For on-cartridge RT-qPCR, 45 cycles of PCR were performed, which allowed us to see the trend of amplification with a low copy number of SARS-CoV-2 targets. A cycle threshold value less than 40 is interpreted as positive for SARS-CoV-2 detection.

### 2.3. Microfluidic-Based Integrated Detection-Analysis System—MiDAS Instrument

**Figure 1:**
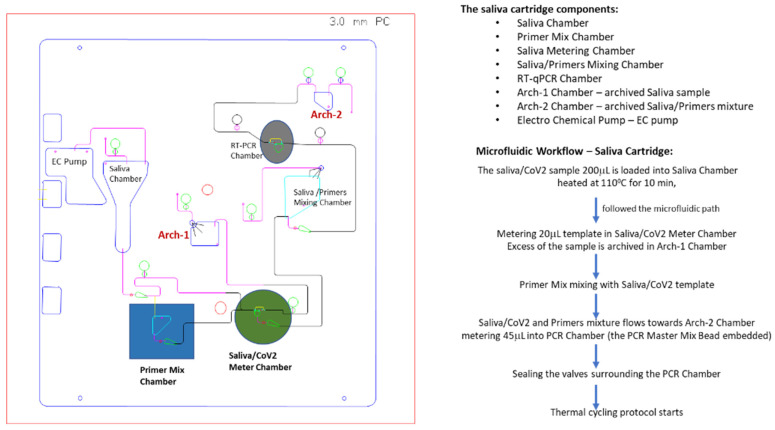
Schematic illustration of SARS-CoV-2 RT-qPCR cartridge layout. A saliva cartridge is composed of several chambers (saliva chamber, primer mix chamber, saliva/SARS-CoV-2 meter chamber, saliva/primers mixing chamber, RT-PCR chamber, and archive chambers), connecting channels, valves, and pumps. Each functional chamber is labeled corresponding to its content. The saliva cartridge microfluidic workflow and volumes are outlined and indicated.

Using the existing layout of the control system in the MiDAS benchtop instrument previously reported [33,34], we designed a cartridge to perform a direct lysis of saliva and a multiplex RT-qPCR readout. As in other applications [33,34], we adapted the swab-lysis chamber design for saliva input and added a resistive heating element for the reaction chamber. The cartridge was made of polycarbonate (PC) material and consisted of CNC-machined channels, fluidic chambers, and valves that can completely isolate the sample from the outside environment to drastically reduce the risk of cross-contamination. The input saliva sample volume was 200 µL and heated at 100 °C for 10 min. Only one tenth of the heated saliva (20 µL) is then metered into a saliva metering chamber and used in the on-cartridge detection assay. The metered 20 µL saliva was mixed with primer mix (N1, N3, and RNP primers) to achieve a saliva-primers mixture which was streamed into the PCR chamber through fluidic metering, where the 1-Step RT-qPCR Master Mix (lyophilized bead) was pre-loaded. There are two archive chambers, archive-1 (Arch-1) for receiving a post-metered saliva sample and archive-2 (Arch-2) that collected the post-PCR chamber metered mixture. Archived samples could be retrieved from the chambers to perform on-benchtop or on-isolated PCR chip evaluations for correlating cartridge performance from the same sample source. All the necessary reagents were pre-loaded onto the cartridge prior to run. The lyophilized Qscript Lyo 1-Step RT-qPCR master mix bead was embedded in the PCR chamber during cartridge assembly. The On-Cartridge processes are illustrated in Figure 1. All cartridges are self-contained and disposed of as biohazard waste.

During the optimization of the on-cartridge sample preparation, the metering of the saliva sample posed a challenge for on-cartridge processing since saliva samples are relatively viscous. At the beginning, saliva metering was often incomplete, due to the bubbles generated during fluid metering of 20 µL saliva sample or partial saliva sample metering due to a siphoning effect. Smoothness and cleanness of the chamber surface is essential for successful saliva metering. The depth of the bypass circuit over the metering chamber, the diameters of the inlet and outlet, and the deburring protocol for the narrow channel structures were all adjusted and fine-tuned to achieve a reproducible and accurate saliva metering.

Functional characterization of each cartridge modular component was evaluated separately. The intra-PCR chamber thermal profile was measured and optimized to meet the PCR thermal protocol requirements. Due to the thermal inertia presented by the PC cartridge substrate, the thermal conductivity had to be taken into consideration. The Peltier temperature had to be set 1 °C to 1.5 °C higher than the PCR thermal cycling protocol, in order to achieve the required thermal profile inside the PCR chamber. The on-MiDAS thermal profile of PCR cycling had a heating rate of 5 °C/s and a cooling rate at 4 °C/s, which is comparable to most commercial PCR thermal cycling instruments.

**Figure 2:**
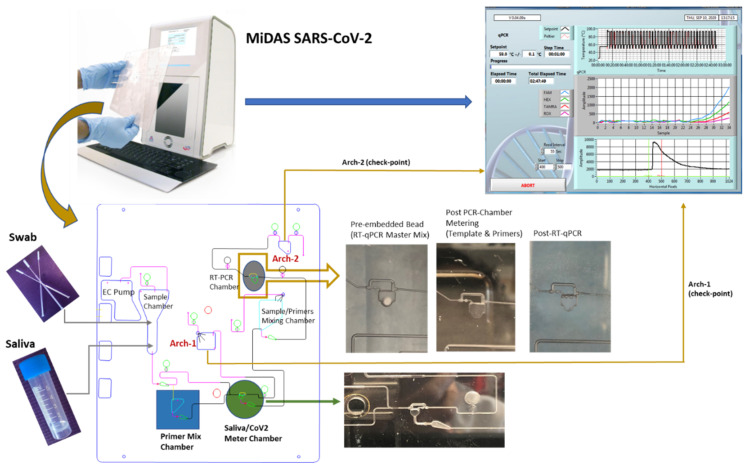
Illustration of the on-MiDAS SARS-CoV-2 detection workflow from saliva sample-in to test result-out. The top panel demonstrates from saliva-in to qPCR result-out. The lower panel details the check points at each functional step during every cartridge in process. The sample chamber can be loaded with saliva or swab specimen. The images show PCR chambers: pre-embedded with lyophilized RT-qPCR master mix bead, post PCR-chamber metering, and post on-cartridge RT-qPCR, and were all taken from a real cartridge. The green arrow points to the image taken from a cartridge with fully metered 20 µL saliva specimen.

Prior to performing on-cartridge RT-qPCR, an isolated PCR chip made of the same PC material as for the cartridge, was used to test detection of SARS-CoV2 RNA and/or γ-irradiation inactivated SARS-CoV-2 virions, as compared to commercial PCR instrument, Stratagene Mx3005 P, with 1-Step Multiplex RT-qPCR Master Mix (MM) of TaqPath (liquid form) and Qscript Lyo bead (lyophilized form) forms. For an easy on-cartridge PCR MM storage and to avoid technical difficulties with loading the liquid form of PCR MM into the PCR chamber before each run, Qscript Lyo 1-Step Multiplex RT-qPCR MM bead (lyophilized pellet) was selected for on-cartridge integration. Figure 2 illustrates the workflow of a saliva cartridge from saliva/CoV2 sample-in or NP swab-in to SARS-CoV-2 test-result-out, along with the check points during the evaluation process.

## 3. Results

### 3.1. MiDAS Instrument, Cartridge Design and Microfluidic Workflow

The design of the saliva cartridge for SARS-CoV-2 detection was based on the assay chemistries workflow (Figure 1). Briefly, 200 µL of saliva/TBE (1:1) sample was spiked with different known copy numbers of either SARS-CoV-2 plasmid-RNA (IDT) or γ-irradiated SARS-CoV-2 virions (BEI, the estimated concentration was 1.7 × 10^10^ virions/mL), and then heated at 100 °C for 10 min, followed by pumping the heat-treated saliva sample through several valves toward the Arch-1 chamber, retaining 20 µL in the saliva metering chamber. The Arch-1 chamber contains the on-cartridge heat-treated saliva sample, which was retrieved after completion of the cartridge run for benchtop evaluation of on-cartridge heat-treatment and mixing efficacies by performing RT-qPCR: (1) on a Stratagene instrument, a benchtop control or (2) in an isolated PCR chip with the same plastic material as the cartridge made of and mixing with Primer Mix to evaluate the on-cartridge mixing efficacy (as a control for on-cartridge primer-saliva mixing). The metered 20 µL sample of saliva/CoV-2 RNA or virions was subsequently mixed with the Primer Mix (80 µL) containing N1-FAM primer-probe (5 µL), N3-ABY primer-probe (5 µL), RNP-VIC primer-probe (2 µL) and nuclease-free water. In the mixing chamber, the sample/primers were “bubble”-mixed for 1 min via continuous pumping, followed by flowing the sample-primers mixture towards the Arch-2 chamber, passing through PCR chamber, where one Qscript Lyo 1-Step RT-qPCR lyophilized bead was embedded and 45 µL mixture of saliva/SARS-CoV-2/primers was retained. The extra amount of the saliva/primer mixture was archived in Arch-2 chamber and later retrieved for trouble-shoot or comparison by performing RT-qPCR on benchtop as check-point control for on-cartridge RT-qPCR evaluation (as illustrated in Figure 2 lower panel). Finally, the on-cartridge assay detection of SARS-CoV-2 directly from saliva samples was reproducibly demonstrated.

### 3.2. On-MiDAS Saliva Cartridge Modular Characterization

**Figure 3:**
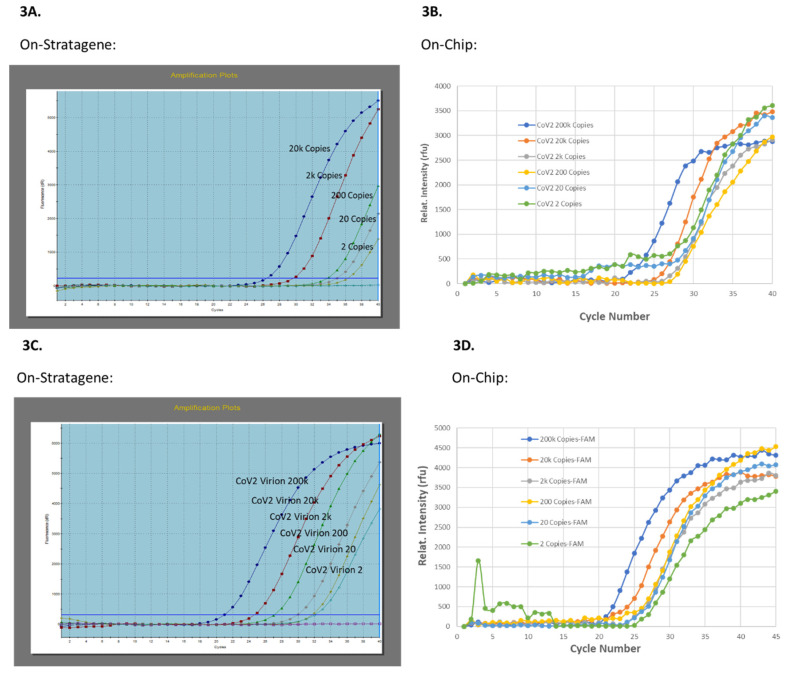
Evaluation of on-MiDAS Peltier thermal cycling unit. Detection of SARS-CoV-2 spiked in saliva was compared between laboratory benchtop equipment (**3A**,**3C**) and on-chip (**3B**,**3D**). 1-Step RT-qPCR was performed with the N1-FAM primer. The viral copy number dose-response curves were generated with either the SARS-CoV-2 plasmid-RNA (**3A**,**3B**) or with γ-irradiated SARS-CoV-2 virions (**3C**,**3D**).

As a benchmark assay control, the TaqPath 1-Step RT-qPCR Master Mix protocol for SARS-CoV-2 detection was selected as the PCR reagent control for the on-cartridge embedded lyophilized bead of Qscript Lyo 1-Step RT-qPCR Master Mix. Several control experiments were also performed and the SARS-CoV-2 RNA copy number dose-response curves (DRC) was generated on (1) laboratory benchtop Stratagene Mx3005 P real-time PCR instrument (Figure 3A,C) and (2) on an isolated PC plastic PCR chip (Figure 3B,D), with both purified SARS-CoV2 plasmid-RNA (Figure 3A,B) and saliva spiked with known copy numbers of γ-irradiated SARS-CoV2 virions (Figure 3C,D). To test the RT-qPCR module on MiDAS system, the same RT-qPCR reagent mixture as the on-Stratagene RT-qPCR, was loaded into an isolated PCR chip (20 µL) and 1-Step RT-qPCR was performed on MiDAS system. Comparable to the benchtop real-time PCR instrument, the on-chip amplification curves were obtained for SARS-CoV2 plasmid-RNA and/or virions in a copy-number dose-response manner, as shown in Figure 3A,B, respectively, validating firstly, the PC plastic material was compatible with RT-qPCR conditions and secondly, the Peltier and detection elements on-MiDAS were matching the requirements for performing 1-Step RT-qPCR.

Before starting to run an integrated sample-in to result-out integrated process, we chose a modular optimization approach to evaluate the function of each module prior to their integration. It included the saliva sample heating time, saliva chamber metering, mixing of metered saliva with primers mix, and intra-PCR chamber thermal profile characterization and adjustment.

### 3.3. Saliva Sample Preparation and Heating Time Evaluation

Saliva samples were collected from healthy individuals, according to the approved IRB protocol. Equal portions of a saliva sample and 1× Tris/Borate/EDTA buffer (TBE, Sigma, St. Louis, MO, USA) were mixed, aliquoted into a 0.2 mL/tube, and spiked with SARS-CoV-2 RNA at 2 × 10^5^, 2 × 10^4^, 2 × 10^3^, 2 × 10^2^, 2 × 10^1^, or 2 × 10^0^ copies/200 µL saliva sample. For SARS-CoV-2 Virions, the saliva sample was spiked with 1 µL of SARS-CoV-2 virions of 2 × 10^8^/mL, 2 × 10^7^/mL, 2 × 10^6^/mL, 2 × 10^5^/mL 2 × 10^4^/mL, or 2 × 10^3^/mL copies into 200 µL saliva aliquots, respectively to achieve final estimated virion copy numbers of 2 × 10^6^, 2 × 10^5^, 2 × 10^4^, 2 × 10^3^, 2 × 10^2^, 2 × 10^1^, or 0 copies/200 µL saliva sample. The samples were heated at 100 °C for 10 min, 5 min, 2 min, 1 min, and 0 min and subsequently 1-Step RT-qPCR was performed on-benchtop with heated samples to generate a heating time courses. The heating time curves of saliva samples spiked with SARS-CoV-2 virions at 200 copies (Figure S1A) and 2 copies (Figure S1B) is presented in the Supplemental Figures. No obvious Ct value shift in different heating times was observed at higher initial template concentration. Without pre-heating (0 min time point), N1-FAM amplification curve was still comparable to those of heated saliva samples, at higher concentration of template (estimated 200 copies and at higher copy numbers). However, this difference in Ct value shift became clear in the pre-heat times when the template concentration was low (estimated two copies). Surprisingly, without pre-heat (heat time = 0 min), SARS-CoV-2 was still detected with PCR cycling. Considering the material mass and the slower heat transfer of plastic cartridges compared to regular PCR tubes, the heating time of 10 min was selected for forward on-cartridge experiments.

### 3.4. On-Cartridge Mixing Module Optimization

Prior to the heated saliva sample reaching the PCR chamber, it needs to mix with the primers. A portion of the saliva (20 µL) and primers (multiple primers, 80 µL) mixture was metered into the PCR chamber where a Qscript Lyo 1-Step RT-qPCR master mix bead is embedded. Sufficient saliva/primers mixing is critical so that each primer’s concentration metered in the PCR chamber, along with the SARS-CoV-2 RNA or virion template, and is proportional, based on the optimal multiplex qPCR protocol generated in the in-tube PCR. In order to evaluate the on-cartridge mixing module, two check-point controls were performed with retrieved Arch-1 and Arch-2 samples. First, 1-Step RT-qPCR was run on an isolated PCR chip (containing Qscript Lyo PCR master mix bead) with the sample retrieved from archive-2 (Arch-2) chamber, which contained the same mixture of saliva and primers as that metered in the cartridge PCR chamber. If the on-chip RT-qPCR with Arch-2 sample failed, the on-chip RT-qPCR with the sample retrieved from archive-1 (Arch-1) chamber (heated saliva/SARS-CoV-2 sample) of the same cartridge could then be tested by mixing the Arch-1 sample on benchtop with the same primer mix as that loaded on the cartridge to perform on-chip 1-Step RT-qPCR. Since the isolated PCR chip was made with the same plastic material and dimensions as the cartridge PCR chamber, performing on-chip PCR could rule out the possibility of a failed on-cartridge PCR being due to the cartridge PCR module, rather than due to insufficient on-cartridge mixing. If the on-chip PCR failed with the Arch-2 sample but not with the Arch-1 sample, it indicated that insufficient on-cartridge mixing was the issue. Following several rounds of design revision and flow-sequence tuning, the on-cartridge “bubble-mixing” was implemented and replaced the “flow-through mixing”. Successful multiplex 1-Step RT-qPCR was achieved not only from on-cartridge but also with the sample retrieved from the Arch-2 chamber, indicating that the improved on-cartridge mixing of sample and primers was the key factor for on-cartridge direct detection of SARS-CoV-2 in saliva samples (Supplementary Materials Figures S2 and S3).

### 3.5. On-MiDAS Evaluation of 1-Step Multiplex RT-qPCR in an Isolated PCR Chip

**Figure 4:**
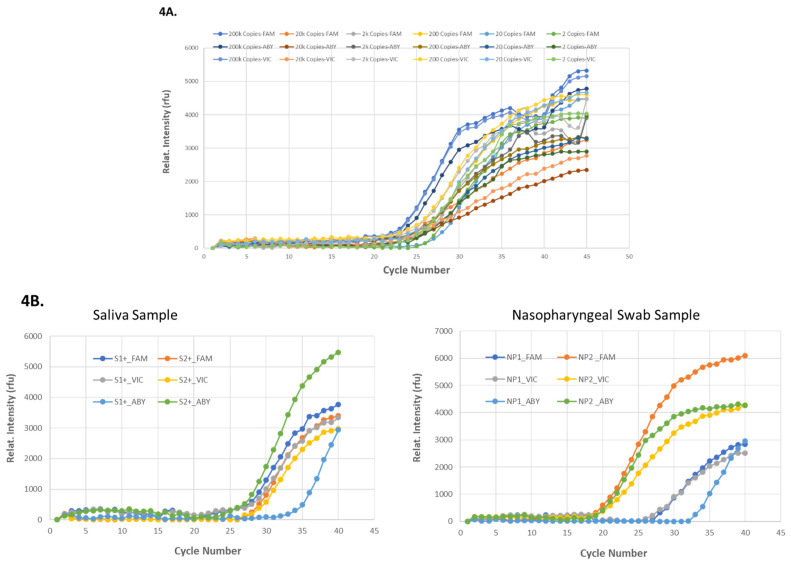
On-chip 1-step multiplex RT-qPCR on-MiDAS. The on-chip 1-step multiplex RT-qPCR with N1-FAM, N3-ABY, and RNP-VIC primers was tested with templates of different concentrations (copy numbers) of 2019-nCoV N Plasmid-RNA (**4A**) and the purified SARS-CoV-2 RNA of unknown concentration from clinical patients’ saliva and nasopharyngeal swab specimens (**4B**). Notations of S1+ and S2+ are saliva specimens (**4B left panel**), NP1 and NP2 are nasopharyngeal specimens (**4B right panel**). N1 primer is labeled with FAM, N3 is labeled with ABY, and RNP is labeled with VIC.

Multiplex 1-Step RT-qPCR of saliva sample spiked with SARS-CoV-2 RNA was first demonstrated in the isolated PCR chip which was run on the MiDAS instrument prior to its implementation in on-cartridge workflow. A triplex RT-qPCR detection of SARS-CoV2 in saliva samples spiked with viral RNA or from some clinical saliva and NP swab samples (WREN Laboratory) were demonstrated, as shown in Figure 4. SARS-CoV2 plasmid-RNA with known copy number concentrations and the RNA samples purified from patients’ saliva (labeled as S1+ and S2+) and NP swabs (labeled as NP1, NP2) with unknown copy number concentrations (WREN Laboratories) were tested for on-chip multiplex 1-Step RT-qPCR on the MiDAS. Triplex 1-Step RT-qPCR with N1-FAM, N3-ABY, and RNP-VIC primers were used and a template concentration (copy number)-dependent of SARS-CoV2 viral RNA amplifications is summarized in Figure 4A. With unknown viral load in the patient samples, SARS-CoV2 was also detected by on-chip 1-Step Multiplex RT-qPCR (Figure 4B), demonstrating 1-Step Multiplex RT-qPCR could be achieved with the PC plastic PCR chips on MiDAS and with good detection sensitivity.

### 3.6. On-Cartridge Direct Detection of SARS-CoV2 from Saliva Samples

After characterizing each of the functional microfluidic modules (see methods), a front-to-end integrated saliva cartridge for direct detection of SARS-CoV-2 virus from saliva samples was tested on MiDAS. Known amounts of SARS-CoV-2 viral RNA or virions were spiked in saliva samples and loaded in the saliva chamber (Figure 1). The SARS-CoV-2 viral copy numbers targeted for the on-cartridge PCR chamber was calculated based on each microfluidic step dilution and volume metered in PCR chamber (Figure S4). Table 1. identifies the relationship between the copy numbers of SARS-CoV-2 RNA spiked in saliva samples loaded into the cartridge’s saliva sample chamber and the copy numbers of SARS-CoV-2 ending up in the PCR chamber for amplification. The sensitivity of the on-cartridge 1-Step Multiplex RT-qPCR on MiDAS instrument was demonstrated with a capability of detecting as low as 9 copies of SARS-CoV-2 viral RNA.

**Figure 5:**
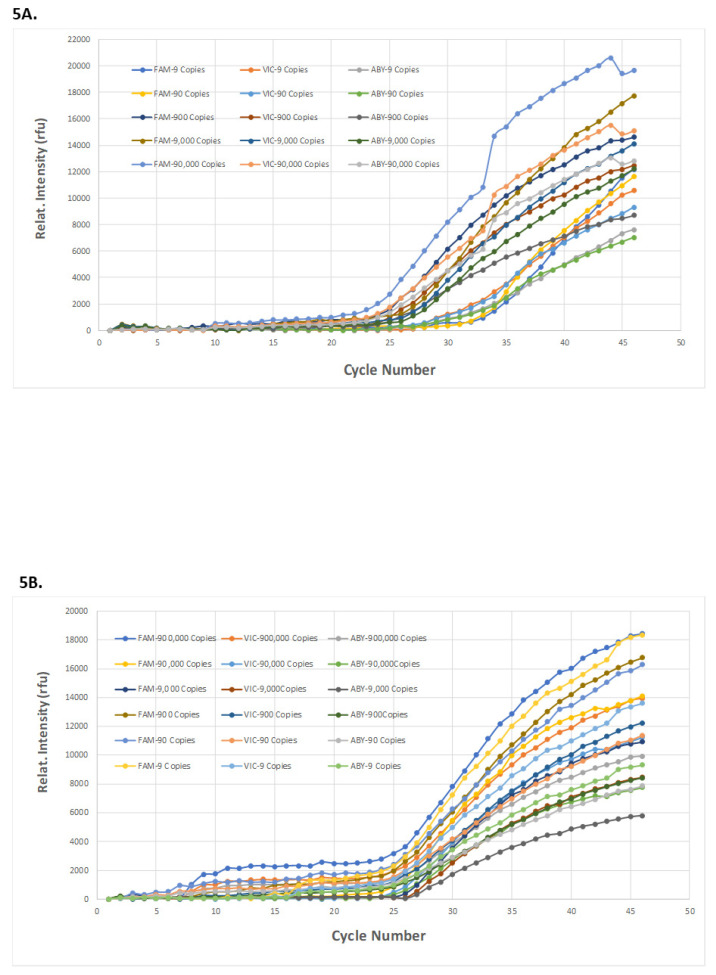
On-cartridge direct detection of SARS-CoV-2 from saliva from sample-in to result-out. Saliva samples spiked with a known copy number of either SARS-CoV-2 plasmid RNA (**5A**) or γ-irradiated SARS-CoV-2 virions (**5B**) were loaded in the saliva chamber of the cartridge. The results from the on-cartridge 1-step multiplex RT-qPCR with N1-FAM, N3-ABY, and RNP-VIC primers are shown. The copy number dose-response curves were generated with purified 2019-nCoV N Plasmid-RNA (**5A**) or γ-irradiated SARS-CoV-2 virions (**5B**).

As outlined in Figure 2, a complete on-cartridge assay on-MiDAS, with “saliva-in and result-out” was demonstrated, detecting SARS-CoV-2 from the saliva sample spiked with either purified SARS-CoV-2 RNA or SARS-CoV-2 virions. One Qscript Lyo 1-Step Multiplex RT-qPCR bead was embedded into the cartridge PCR chamber during cartridge assembly. The well-mixed saliva/CoV2 RNA/primers mixture was directed toward Arch-2 chamber with 45 µL metered into the PCR chamber. The on-cartridge RT-qPCR started, with real-time monitoring after the valves surrounding the PCR chamber were closed. Different concentrations of SARS-CoV2 RNA or virion spiked in saliva were examined for on-cartridge detection. The on-cartridge detection sensitivity and multiplexity were presented in Figure 5A with purified 2019-nCoV-2 Plasmid-RNA and Figure 5B with γ-irradiated SARS-CoV-2 Virions. Triplex PCR amplification curves were generated from a template concentration of 900,000 copies to nine copies of SARS-CoV2 RNA and virions. Sample retrieved from the cartridge Arch-2 chamber, which contains on-cartridge mixed saliva and primers, were checked on a benchtop real-time PCR system to further evaluate the on-cartridge mixing and multiplexing PCR (Figure S5). Comparing the on-cartridge RT-qPCR amplification result (Figure S5A) with the RT-qPCR result from the Arch-2 sample retrieved from the same cartridge (Figure S5B), the amplification intensity obtained from the Arch-2 sample was often seen to be attenuated. This can be attributed to a time lag between retrieving the sample from the Arch-2 chamber and running the sample as an RT-qPCR control. The efficacy of PCR reagents in the mixture retrieved from the cartridge would decrease before the RT-qPCR control was performed. The Qscript Lyo 1-Step Multiplex RT-qPCR bead was used in both on-cartridge and in-tube detections.

## 4. Discussion and Conclusions

The MiDAS SARS-CoV-2 detection system offers a sample-to-answer nucleic acid amplification test, which can yield a diagnostic result from saliva specimens in less than 2 h, comparable to a benchtop commercial real-time RT-PCR instrument (Stratagene Mx3005P), with a triplex amplification of two SARS-CoV-2 target genes (N1 and N3) and one RNP gene as internal control. The input saliva sample spiked with SARS-CoV-2 virions was as low as 1000 copies/mL or 1 copy/µL tested. The output detection sensitivity demonstrated by real-time RT-PCR was as low as 200 copies/mL (Table 1).

In response to the SARS-CoV-2 pandemic, one of the biggest challenges is the ability to reliably provide rapid and accurate diagnostic test results. High quality specimen collection, storage and transport are the first steps in ensuring the accuracy of testing. The gold standard test for SARS-CoV-2 infection was considered to be real-time RT-PCR of upper respiratory tract specimens, i.e., NP or OP swabs, or bronchoalveolar lavage. Due to the invasiveness of the procedure, most people experience discomfort during swabbing and this collection method therefore is associated with high variations in accuracy. The process itself puts healthcare workers at potential risk of exposure. Subsequently, saliva testing has become more and more popular as a sampling method for diagnostic purposes [10,20]. Saliva sample collection has several advantages over NP swabs; such as ease of self-collection, non-invasive home sampling, and it can capture high viral loads [13,35]. Many studies have reported saliva as an important mediator in transmitting SARS-CoV-2 virus through coughing, sneezing, droplets and aerosols [36,37]. In addition, direct RT-qPCR of heated saliva samples without an RNA extraction step can shorten the assay time, use less sample (volume), and thus alleviate reagents shortage making it suitable for general screening [15,16,17,18,19,20]. Recently, detection of SARS-CoV-2 was compared between saliva and NP or OP swab samples [14]. This meta-analysis conclusively demonstrated that saliva was as valid as NP sampling for the detection of SARS-CoV-2 infection in both symptomatic and asymptomatic carriers.

In our study, we adapted our previously reported [33,34] portable MiDAS system, to undertake SARS-CoV-2 molecular testing using saliva samples (Figure 1 and Figure 2). With no lysis buffer added but using direct on-cartridge heating, a limit of detection of SARS-CoV-2 virions at 1000 copies/mL of input saliva sample was achieved, resulting in an on-cartridge qPCR sensitivity for SARS-CoV-2 at nine viral copies in 45 µL volume (Table 1). Considering the chamber material mass and thermal transfer property of the plastic cartridge, a higher temperature (set at 110 °C) and a longer heating time (10 min, the longest heating time tested Figure S1) were used for the on-cartridge assay parameters to ensure that the temperature inside the saliva chamber reached boiling temperature and that viral lysis occurred during on-cartridge heating. Although the input concentration of viral particles per mL saliva was similar to the detection limit reported on-benchtop [18], our on-cartridge detection showed higher sensitivity of approximately 1000 viral particles per mL in a triplex RT-qPCR, meeting the CDC requirement for SARS-CoV-2 detection with an RT-qPCR assay. Compared to SalivaDirect^TM^, which is also a nucleic acid extraction-free method developed for direct detecting SARS-CoV-2 virions from saliva, our assay platform did not require addition of proteinase K for the lysis of saliva sample before heating. The reported detection limit in this assay was 6–12 copies/µL [20] in an on-benchtop duplex RT-qPCR. In comparison, our on-cartridge RNA-extraction-free detection limit reached as low as 1000 copies/mL (nine copies/45 µL), in a triplex RT-qPCR, 30–60 times more sensitive.

In a collaborative effort to limit the community spread of SARS-CoV-2, it is critical to easily access rapid and accurate testing. As a result, many point-of-care (POC) devices are under EUA for the rapid diagnosis of COVID-19. For example, CovidNudge, an integrated lab-on-chip device can process OP or NP swab specimens in 90 min, from swab sample-in to PCR result out [26]. Different from our MiDAS SARS-CoV-2 system, its sample preparation unit extracts RNA first from swab samples, which is followed by target amplification in a 72-well plate format. The advantage of the CovidNudge platform is that it allows amplification of seven target genes per sample and can run multiple (6–9) replicates to increase the test reliability. Although it demonstrated an overall sensitivity of 94% with SARS-CoV-2 positive NP or OP swab samples in this diagnostic accuracy study [26], as compared to the standard laboratory on-bench RT-qPCR, the viral load in the specimen was unknown. The Abbott Diagnostics ID NOW was reported to be a POC isothermal amplification-based platform that detects SARS-CoV-2 in approximately 17 min, with a detection limit of 2000 copies/mL of saliva specimens [38]. The Cepheid Xpert Xpress SARS-CoV-2 device is also a cartridge-based assay platform for NP swab specimens, with a limit of detection of 250 copies/mL as provided by the manufacturer and 100 copies/mL as reported by other recent evaluations [39]. The explanation for the discrepancy in limit of detection from different institutional studies is most likely due to the different methods used for determining input concentrations. Although a limit of detection was as low as 8.26 copies/mL with single target amplification, the Ct values of 39.8 and 42.2 were reported by a multi-center evaluation of Xpress SARS-CoV-2 system [40], which reaches or surpasses the cutoff limit for positive calling considered by most PCR-based bioassays. Additionally, only one target gene was amplified. Another sample-to-answer nucleic acid amplification device, the Accula SARS-CoV-2 POCT (Mesa Biotech), was evaluated with 100 clinical NP swab specimens and read out via lateral flow [41]. Comparing to a reference EUA SARS-CoV-2 Laboratory Developed Test (LDT), the Accula POCT showed low diagnostic accuracy at low viral load, although it provides potential advantage of rapid POCT within 30 min. We took a different approach by detecting viral particles from saliva specimens directly, bypassing the viral RNA extraction step and performing multiplexed qPCR. Moreover, the cost for PCR-based molecular test using the commercially available POC instruments could be high for those un-insured patients (Supplementary Material Table S1). For on-MiDAS SARS-CoV-2 detection, the cost per saliva sample was around $13 per cartridge.

Similar to our MiDAS SARS-CoV-2 system, many research institutions have also been actively involved in developing portable POC devices for COVID-19 detection. One of those examples is Mobilefuge, a 3D-printed centrifuge device which was designed for viral RNA isolation and could be powered by mobile phones [32]. However, it is not a sample-to-answer device yet. The Loop-Mediated Isothermal Amplification (LAMP) module is not integrated and no actual result from viral RNA isolation was demonstrated. A CRISPR-based POC diagnostic, miSHERLOCK platform was reported to detect SARS-CoV-2 variants in one hour from saliva samples [25] with a LOD of 1000 copies/mL achievable. Although reported as a minimally instrument, several user hands-on steps are required; for example, adding hazardous chemicals (DTT and lysis buffer) to the saliva sample, a manual transfer of the flow columns into the reaction chamber, and depressing the plunger to release the RNA-capture membrane.

The MiDAS system was adapted for SARS-CoV-2 test used micromachined cartridges which will be optimized for better reproducibility by scale-up manufacturing and injection molding. Although we demonstrated the sensitivity and accuracy of direct detection of SARS-CoV-2 from saliva samples spiked with known copy number of SARS-CoV-2 RNA or virions, further tests with a larger set of clinical samples is also necessary to validate the system for clinical application. With the scalability of this platform, an effort for on-cartridge simultaneous processing of higher throughput of specimen in multiple cartridges is in progress.

Herein, we report, a microfluidic cartridge-based sample-to-answer POC device adapted for SARS-CoV-2 detection, directly from self-collected saliva specimens. The SARS-CoV-2 test is processed in a sealed cartridge, which is inserted into the MiDAS instrument after the saliva sample is loaded in the sample chamber, with minimal hands-on time. As the genetic variants of SARS-CoV-2 have emerged over the course of the COVID-19 pandemic, the need for readily accessing reliable, automated and sensitive virus detection in outpatient settings is further emphasized. Monitoring emerging SARS-CoV2 variants requires a global effort to ensure diagnostic tests, vaccines, and other antiviral therapies remain effective. Currently, over 1.2 million viral genome sequences have been collected from 172 countries and are available to track the SARS-CoV2 evolution and global spread. Development of preventive measures against the mutating virus continues to increase the demand for faster PCR-based workflows. MiDAS SARS-CoV-2, a portable Microfluidic-based Integrated Detection-Analysis System, contributes to the development of a sensitive and accurate multiplex COVID-19 detection. Although further evaluation with a large test sample size is necessary, we have demonstrated a direct detection of SARS-CoV2 virus from saliva samples, with an on-cartridge PCR sensitivity of as low as nine copies in the reaction volume. The use of a lyophilized RT-qPCR master mix alludes to a high stability of the reagents even when pre-embedded on the cartridge. The use of a disposable dedicated plastic cartridge avoids sample-to-sample cross-contamination. With its portability, high sensitivity, automation capability, and low cost, the MiDAS SARS-CoV-2 system can be used in field testing and areas/countries with poor resources. The potential of scaling up production for multiple sample input and parallel processing can benefit wide public health screenings, for example school campus-wide monitoring programs, back-to-work screen programs, and global viral surveillance.

## Figures and Tables

**Table 1:** On-cartridge detection of SARS-CoV-2 directly from saliva samples, input vs. output.

SARS-CoV-2 Viral RNA Copy Number	SARS-CoV-2 Viral RNA Copy Number
Spiked in 200 mL Saliva-TBE (1:1) Sample	in the RT-PCR Chamber (in Volume of 45 mL)
2,000,000	90,000
200,000	9000
20,000	900
2000	90
200	9

On-cartridge SARS-CoV-2 detection with either purified SARS-CoV-2 RNA or SARS-CoV-2 virions. The left column shows the input of the saliva specimen containing different concentrations (copy number) of SARS-CoV-2 RNA/virions, from 2,000,000 copies down to 200 copies of RNA or viral particles loaded into the cartridge sample chamber, The right column shows the SARS-CoV-2 RNA copy numbers in the PCR chamber (45 µL) amplified.

## Data Availability

All the data and materials used in this study have been mentioned in the article.

## Supplementary Materials

The following are available online at https://www.mdpi.com/article/10.3390/idr13040097/s1, Figure S1: Heating time course of saliva samples spiked with SARS-CoV-2 virions. Figure S2: Evaluation of the earlier design of cartridge with flow-through mixing module. Figure S3: Evaluation of on-cartridge “bubble” mixing module. Figure S4: A simplified diagram showing on-cartridge microfluidic dilution factors. Figure S5: One example of on-cartridge SARS-CoV-2 virion detection from saliva. Table S1: A brief cost comparison of major FDA-approved POC instruments commercially available for SARS-CoV-2 diagnostic test.
