# Supplementary material for: A Sensitive, Portable Microfluidic Device for SARS-CoV-2 Detection from Self-Collected Saliva
Source: Infect Dis Rep. 2021 Dec 14;13(4):1061–77. doi: 10.3390/idr13040097 (PMC8701218; doi:10.3390/idr13040097)
Supplement: Supplementary file 1 [file idr-13-00097-s001.zip › idr-1491791-supplementary.pdf]

Supplementary Materials for:

A sensitive, portable microfluidic device for SARS-CoV-2 detection from self-collected saliva

Jianing Yang[1], Mark Kidd[2], Alan Nordquist[1], Stan Smith[1], Cedric Hurth[1] Irvin M. Modlin[2], and Frederic Zenhausern[1]

[1]Center for Applied NanoBioscience-Medicine, University of Arizona, United States of America, [2]WREN Laboratories, Branford, Connecticut, United States of America.

Insert figure

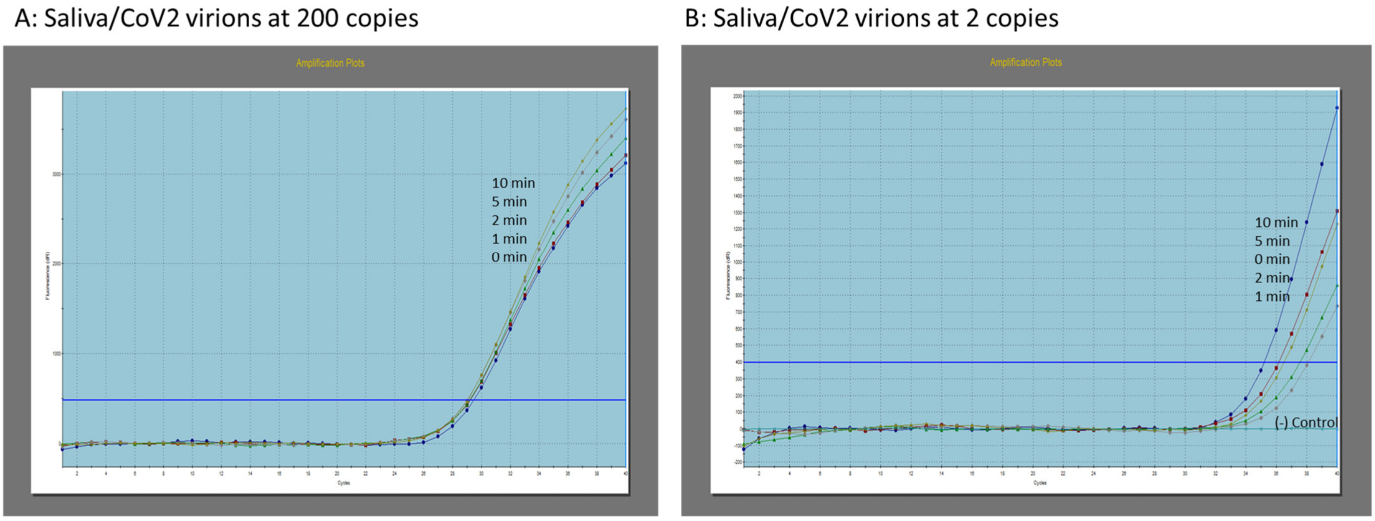

Figure S1. Heating time course of saliva samples spiked with SARS-CoV-2 virions. Saliva samples spiked with SARS-CoV-2 virions at 200 copies (A) and 2 copies (B) were heated at 100°C for 10min, 5min, 2min, 1min, and 0min (no heating). The 1-Step RT-qPCR with N1 primer was performed on benchtop (Stratagene Mx3005P) and the amplification curves were plotted.

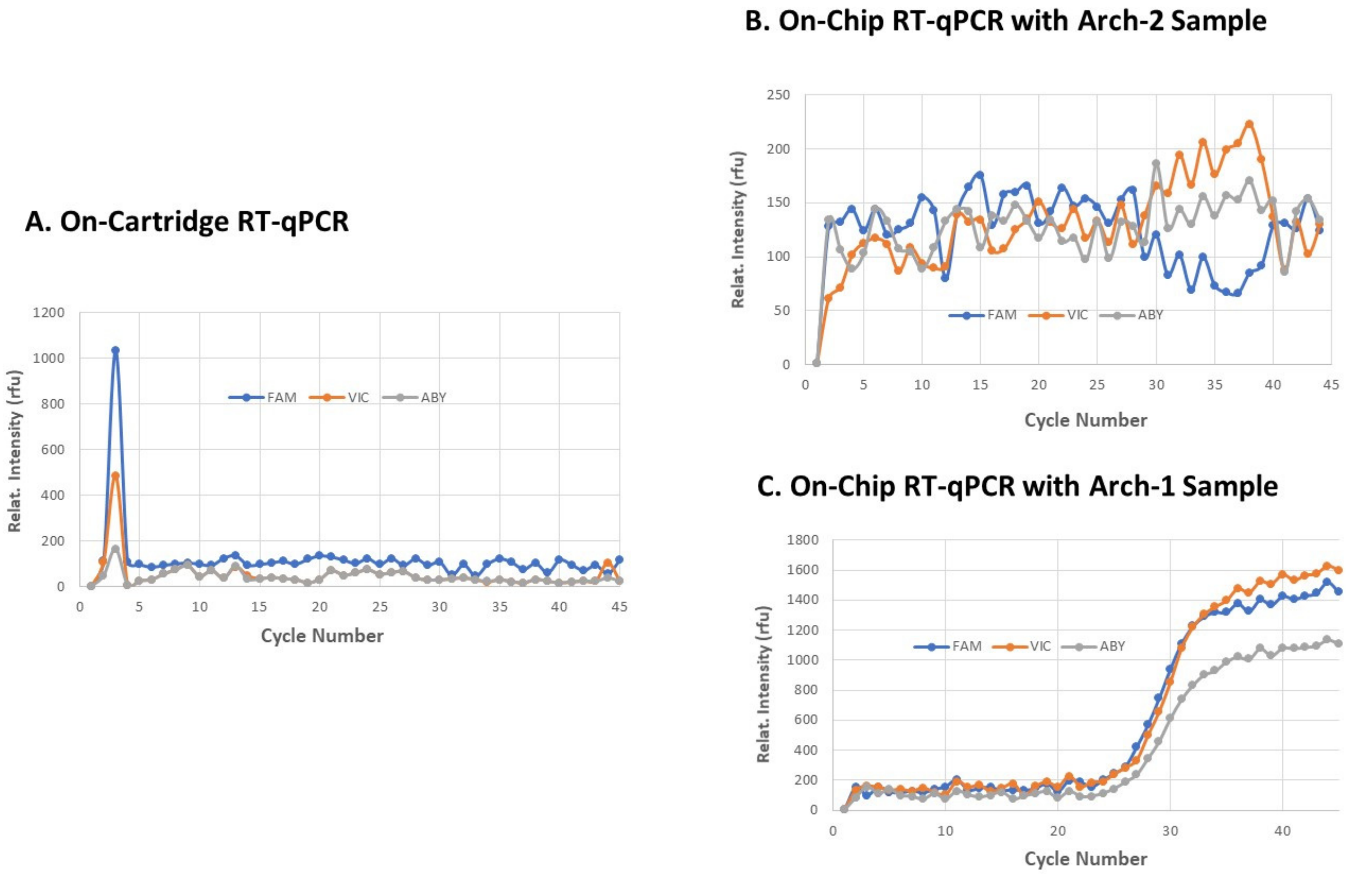

Figure S2. Evaluation of the earlier design of cartridge with flow-through mixing module. Saliva sample was spiked with SARS-CoV-2 virions 9000 copies and processed on the saliva cartridge. The results of 1-step triplex, RT-qPCR with N1-FAM, N3-ABY, and RNP-VIC were presented. The microfluidic flow sequence was: saliva sample from Saliva/CoV2 Meter Chamber (20µL of heated sample) flowing through → Primers Mix Chamber, picking up the primer mix (80µL) and ending at → Saliva/Primers Mixing Chamber, diffusion-mix for 1min, then flowing toward PCR Chamber → RT-PCR Chamber metering (45µL) and the excess of saliva/primers mixture was archived in Arch-2 chamber. The failed on-cartridge RT-qPCR (A) was evaluated by on-chip RT-qPCR with samples retrieved from Archive-2 (B) and Archive-1 chambers (C) of the same cartridge. No amplification was achieved from the on-chip 1-Step RT-qPCR with Archive-2 sample which contained a mixture of on-cartridge flow-through mixed saliva and primers. A successful on-chip 1-Step RT-qPCR was achieved with Archive-1 sample, in which the on-cartridge heated saliva sample was mixed with primers in tube before loaded onto the PCR chip, indicating the insufficient on-cartridge sample/primers mixing was the cause for failed on-cartridge PCR.

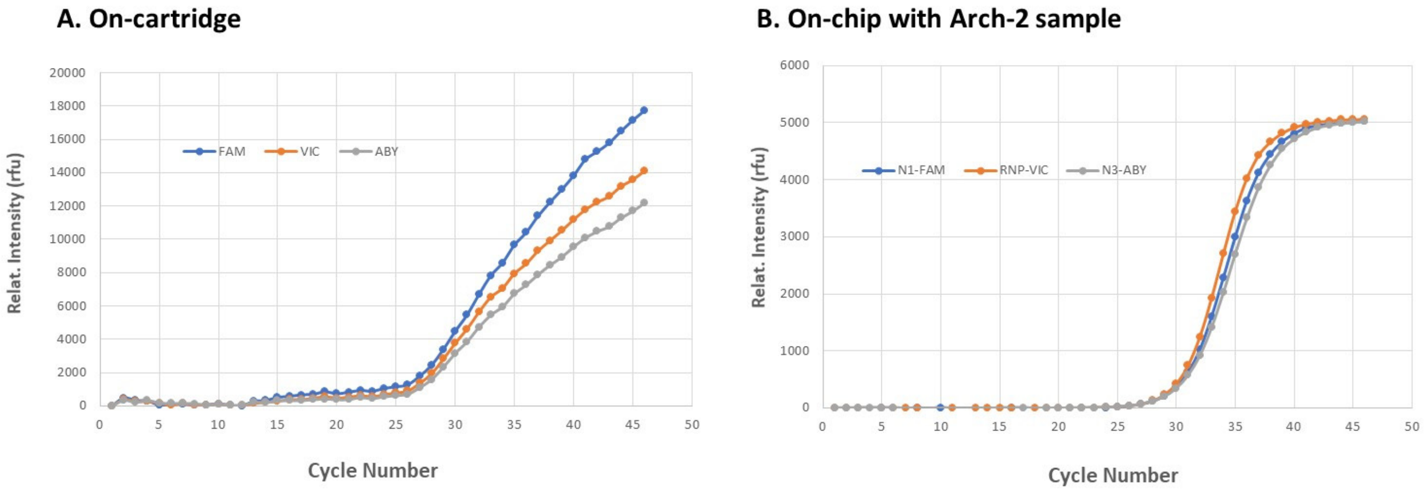

Figure S3. Evaluation of on-cartridge "bubble" mixing module. After several rounds of modification and testing, the on-cartridge flow-through mixing was replaced with "bubble" mixing. The microfluidic flow sequence was: primer mix (80µL) from Primers Mix Chamber flowing through → Saliva/CoV2 Meter Chamber (20µL of heated sample) and ending at → Saliva/Primers Mixing Chamber and bubble-mixing for 1min, then flowing toward PCR Chamber → RT-PCR Chamber metering (45µL) and the excess of saliva/primers mixture was archived in Arch-2 chamber. SARS-CoV-2 RNA 9000 copies were spiked into the saliva sample. After the on-cartridge "bubble" mixing, successful multiplex RT-qPCR amplification was achieved in both on-cartridge (A) and on-chip with sample retrieved from Arch-2 chamber (B).

The amplification intensity was lower in the on-chip RT-qPCR from the Arch-2 sample, which was attributed to time lag between the sample retrieval from the Arch-2 chamber and running the RT-qPCR control. In general, the archived samples were retrieved from the cartridges and stored at -20^{0}C till needed to test for on-chip RT-qPCR on the same MiDAS instrument.

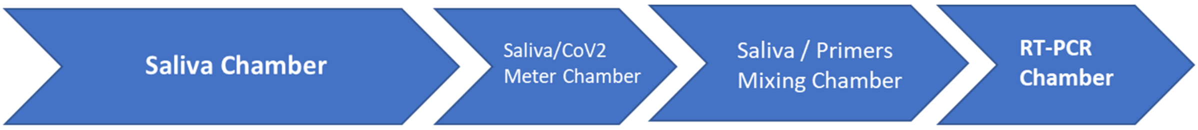

Chamber Name	Saliva Chamber	Saliva/CoV-2 Meter Chamber	Saliva / Primers Mixing Chamber	RT-PCR Chamber
Volume	Input Saliva 200µL	Metered Saliva 20µL	Saliva-Primers Mix 20µL + 80µL	Metered Saliva-Primer Mix 45µL
[target] Dilution		1/10th of Saliva/CoV-2	x5 dilution	½ of Saliva/Primers Mixture
Example (copy#)	2000 copies	200 copies	200 copies in 100µL	90 copies in 45µL

Figure S4. A simplified diagram illustrating on-cartridge microfluidic dilutions of the input target. The target concentration in the initial input sample was different from the target concentration ended in the RT-PCR chamber, due to the dilution and volume changes. During on-cartridge process, only 20µL (one tenth) of the heated saliva sample was metered in the Saliva/CoV-2 Meter Chamber and mixed with 80µL of Primer Mix (five-fold dilution), then only 45% of the mixture from Saliva/Primer Mixing Chamber was metered in the RT-PCR Chamber for on-cartridge 1-Step Multiplex RT-qPCR. Therefore, the system could detect 1000 copies/mL of SARS-CoV-2 virions in saliva samples and the on-cartridge RT-qPCR could detect as low as 9 copy /45µL.

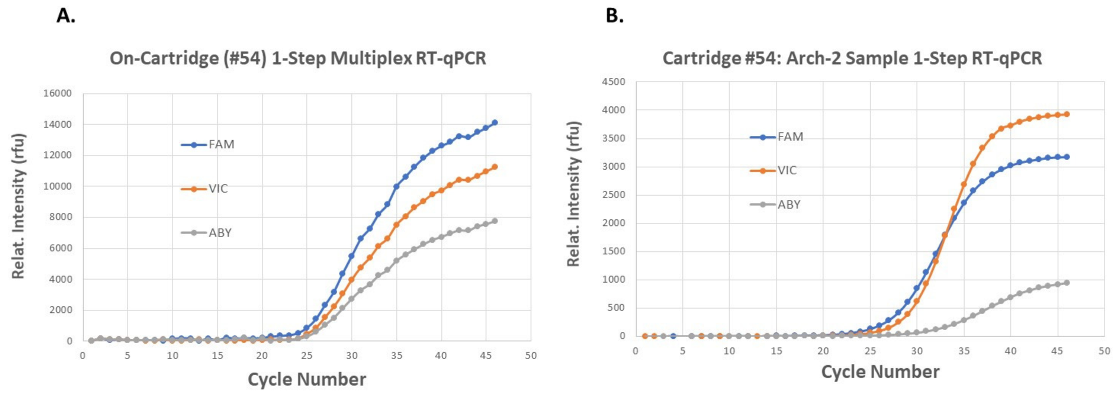

Figure S5. One example of on-cartridge SARS-CoV-2 virion detection from saliva. The Arch-2 samples retrieved from the same cartridge were routinely checked for multiplex 1-step RT-qPCR on an isolated PCR chip. The overall amplification intensity for all three primer sets was decreased in the retrieved Arch-2 samples (B) as compared to the on-cartridge amplification (A). This was attributed to time lag between the sample retrieval from the Arch-2 chamber and running the RT-qPCR control. The efficacy of PCR reagents in the mixture retrieved from Arch-2 would decrease before running the on-chip RT-qPCR control. Samples retrieved from Arch-2 chambers, which contained both saliva/CoV-2 template and primers, were stored at -20°C and RT-qPCR control runs were performed at a later time.

Table S1. A brief cost comparison of major FDA-approved POC instruments commercially available for SARS-CoV-2 diagnostic test

POC Equipment	Sample Type	Turnaround Time	Cost per Test ($)	Resources for The Cost
Abbott ID NOW (Abbott)	saliva	20 min	43	https://www.sdbor.edu/mediapubs/Documents/Abbott%20ID%20Now%20Universities%20TRS%202020.pdf
Xpert Xpress SARS-CoV-2 Device (Cepheid)	NP swab	20 min	20	https://www.msf.org/diagnostic-company-cepheid-charging-more-it-should-covid-19-tests
Accula SARS-CoV-2 POCT (Mesa Biotech)	NP swab	30 min	20	https://cen.acs.org/analytical-chemistry/diagnostics/Rapid-COVID-19-testing-breaks/98/web/2020/08
Mobilefuge	Saliva	n/a*	n/a	Reference [32]
Sherlock	saliva	55 - 120 min	n/a	Reference [25]
WREN Laboratory	NP swab / saliva	24 - 48 hours	80-100	https://www.wrencovidtesting.com
Urgent Care / Clinical Lab test	NP swab	24 - 72 hours	100 - 200	How to shop for FDA-authorized home Covid test kits: A guide (nbcnews.com)
At-home Test / Shipping to Test Centers	NP swab / Saliva	24 - 72 hours	109 - 155	How to shop for FDA-authorized home Covid test kits: A guide (nbcnews.com)
Emergency Rooms	NP swab	varies	varies	
* n/a = not available				
